# Spasmolytic Activity of 1,3-Disubstituted 3,4-Dihydroisoquinolines

**DOI:** 10.3390/biomedicines12071556

**Published:** 2024-07-13

**Authors:** Miglena Milusheva, Mihaela Stoyanova, Vera Gledacheva, Iliyana Stefanova, Mina Todorova, Stoyanka Nikolova

**Affiliations:** 1 Department of Organic Chemistry, Faculty of Chemistry, University of Plovdiv, 4000 Plovdiv, Bulgariastoyanova@uni-plovdiv.bg (M.S.); minatodorova@uni-plovdiv.bg (M.T.); 2 Department of Bioorganic Chemistry, Faculty of Pharmacy, Medical University of Plovdiv, 4002 Plovdiv, Bulgaria; 3 Department of Medical Physics and Biophysics, Faculty of Pharmacy, Medical University of Plovdiv, 4002 Plovdiv, Bulgaria; vera.gledacheva@mu-plovdiv.bg (V.G.); iliyana.stefanova@mu-plovdiv.bg (I.S.)

**Keywords:** muscle relaxant activity, antioxidant activity, isoquinolines, in silico, Bischler–Napieralski reaction

## Abstract

This article concerns the spasmolytic activities of some novel 1,3-disubstituted 3,4-dihydroisoquinolines. These compounds can be evaluated as potential therapeutic candidates according to Lipinski’s rule of five, showing high gastrointestinal absorption and the ability to cross the blood–brain barrier, which is a very important parameter in the drug discovery processes. In silico simulation predicted smooth muscle relaxant activity for all the compounds. Since smooth muscle contractile failure is a characteristic feature of many disorders, in the current paper, we concentrate on the parameters of the spontaneous contractile responses of smooth muscle (SM) cells compared to the well-known drug mebeverine. Two of the newly synthesized substances can be identified as essential modulating regulators and potentially used as therapeutic molecules. One of these molecules also showed significant DPPH antioxidant activity compared to rutin.

## 1. Introduction

The isoquinolines are a family of phytochemicals found in plants, like Papaveraceae, Berberidaceae, and Ranunculaceae. The alkaloids found in these plants possess a remarkable number of biological activities. The isoquinoline ring has been found to possess a wide range of biological and pharmacological activities, for example, antimalarial, anti-HIV, insect-growth-retarding, antitumor, antimicrobial, antifungal, antiparasitic and insecticidal, antiviral, anti-inflammatory, and antiplatelet, activities [1,2,3]. The first isolated and reported isoquinoline alkaloid from *Papaver somniferum* at the beginning of the 19th century was morphine. More than 2500 new structures have since been described, turning the isoquinolines into one of the most complex and diverse groups of alkaloids [4,5]. Due to their vast chemical versatility, isoquinolines have been grouped into 13 major subclasses, such as bisbenzylisoquinolines, pavines, cularines, aporphines, promorphinans, protoberberines, berberines, and simple benzylisoquinolines [6]. Papaverine, for example, belongs to the benzylisoquinoline alkaloids. For decades, this compound has been known as a brain and coronary vasodilator and muscle relaxant, and for its non-specific spasmolytic activity [7]. Papaverine has been known for ages because of its spasmolytic activity [8,9,10,11,12,13], but it found applications in the inhibition of mitochondrial metabolism, the enhancement in tumor oxygenation [14,15], HCoV–OC43 treatment [16], etc. Therefore, an isoquinoline can be an important starting point for drug discovery. The wide range of biological activities associated with isoquinolines set our interest in obtaining novel derivatives and establishing a correlation between their structure and activity.

In the search for novel biologically active molecules, we present here a simple synthesis of 1,3-disubstituted 3,4-dihydroisoquinolines. Using previously described amides [17], the current work attempted to synthesize a number of 3,4-dihydroisoquinoline analogs via the Bischler–Napieralski reaction. 

To further understand their biological characteristics, all of these substances were evaluated for their potential spasmolytic activity. Antispasmodics are typically used to reduce excessive smooth muscle spasms, which can be caused by a variety of illnesses and result in stomach cramps and pain. The genitourinary, biliary, or gastrointestinal tract is affected by these spasms. Antispasmodics are used to treat biliary colic and irritable bowel syndrome (IBS) by decreasing the contractility of smooth muscle [18].

## 2. Materials and Methods

All solvents and reagents were purchased from Merck (Merck KgaA, Darmstadt, Germany). The compounds were characterized by their ^1^H-NMR, ^13^C-NMR, and HRESIMS spectra. The purity of the compounds was determined by TLC (precoated 0.2 mm Merck silica gel 60 plates (Merck KgaA, Darmstadt, Germany)). NMR spectra were recorded at room temperature (ca. 295 K) on a Bruker Avance III HD 500 spectrometer (Bruker, Billerica, MA, USA) at 500 MHz.

HRESIMS spectra were acquired in positive mode on a Q Exactive Plus (ThermoFisher Scientific, Inc., Bremen, Germany) mass spectrometer equipped with a heated HESI-II source. Operating conditions for the HESI source used in positive ionization mode were +3.5 kV spray voltage, 320 °C capillary and probe heater temperature, sheath gas flow rate of 36 a.u., auxiliary gas flow rate of 11 a.u., spare gas flow rate of 1 a.u. (a.u. refers to the arbitrary values set by Exactive Tune software 2.4), and S-Lens RF level of 50.00. Nitrogen was used for sample nebulization and as collision gas in the HCD cell. Aliquots of 1 µL of the solutions of the samples (ca. 20 µg mL^−1^) were introduced into the mass spectrometer through the LC system, Thermo Scientific Dionex Ultimate 3000 RSLC (Germering, Germany), consisting of 6-channel degasser SRD-3600, high-pressure gradient pump HPG-3400RS, autosampler WPS-3000TRS, and column compartment TCC-3000RS equipped with narrow-bore Hypersil GOLD™ C18 (2.1 × 50 mm, 1.9 μm) column. Each chromatographic run was carried out isocratically with a mobile phase consisting of water–acetonitrile–methanol–acetic acid (25:50:25:0.2). The solvent flow rate was 300 μL min^−1^. Full MS–SIM was used as MS experiment in negative and positive mode, where the resolution, automatic gain control (AGC) target, maximum injection time (IT), and mass range were 70,000 (at *m*/*z* 200), 3 × 10^6^, 100 ms, and *m*/*z* 100–500, respectively. Xcalibur (Thermo Fisher Scientific, Waltham, MA, USA) ver. 4.0 was used for data acquisition and processing.

### 2.1. Experimental Protocols and Spectral Data

To a solution of 3 mmol of the corresponding amide **4** [17] in 1,2-dichloroethane, 1 mL of phosphorus(V) oxychloride was added. The reaction mixture was refluxed for 1 h at 100 °C, poured in water, then extracted with CH_2_Cl_2_ (3 × 20 mL), and washed with Na_2_CO_3_ and water. The organic layer was dried using anhydrous Na_2_SO_4_, filtered on the short column filled with basic Al_2_O_3_, and then concentrated.

3-isopropyl-1-methyl-3,4-dihydroisoquinoline (**5a**), yellow oil, 62% yield, ^1^H-NMR: 7.26–7.29 (m, 2H), 7.19–7.22 (m, 3H), 4.07 (ddd, 1H, *J* = 9.3, 5.6, 3.7, CH), 2.88 (dd, 1H, *J* = 13.7, 5.4, CH_2_), 2.74 (dd, 1H, *J* = 14.2, 8.8, CH_2_), 1.9 (s, 3H, COCH_3_), 1.82–1.86 (m, 1H, CH(CH_3_)_2_), 1.0 (d, 3H, *J* = 6.8, CH(CH_3_)_2_), 0.98 (d, 3H, *J* = 6.8, CH(CH_3_)_2_); ^13^C-NMR: 171.02, 138.42, 129.16, 128.36, 126.37, 56.46, 37.88, 30.99, 22.37, 19.58, 17.92; HRMS Electrospray ionization (ESI) *m*/*z* calcd. for [M+H]^+^ C_13_H_17_N^+^ = 188.28078, found 188.27036 (mass error ∆m = −1.04 ppm).

3-isopropyl-1-phenyl-3,4-dihydroisoquinoline (**5b**), yellow oil, 70% yield, ^1^H-NMR: 7.61–7.63 (m, 2H), 7.45–7.47 (m, 1H), 7.36–7.40 (m, 2H), 7.18–7.26 (m, 5H), 4.30–4.35 (m, 1H, CH), 2.93–2.97 (m, 1H, CH_2_), 2.81–2.85 (m, 1H, CH_2_), 1.85–1.92 (m, 1H, CH(CH_3_)_2_), 1.03 (d, 3H, *J* = 6.4, CH(CH_3_)_2_), 1.01 (d, 3H, *J* = 6.4, CH(CH_3_)_2_); ^13^C-NMR: 167.28, 138.32, 135.12, 131.23, 129.22, 128.53, 128.47, 126.41, 55.51, 37.98, 30.77, 19.70, 17.84.; HRMS Electrospray ionization (ESI) *m*/*z* calcd. for [M+H]^+^ C_18_H_20_N^+^ = 250.15903, found 250.15866 (mass error ∆m = −1.46 ppm).

1-(2-chlorophenyl)-3-isopropyl-3,4-dihydroisoquinoline (**5c**), yellow oil, 75% yield, ^1^H-NMR: 7.45–7.47 (m, 1H), 7.35–7.36 (m, 1H), 7.29–7.33 (m, 3H), 7.24–7.26 (m, 3H), 4.34–4.42 (m, 1H, CH), 2.96 (dd, 1H, *J* = 14.2, 6.4, CH_2_), 2.79 (dd, 1H, *J* = 14.2, 8.3, CH_2_), 1.88–1.94 (m, 1H, CH(CH_3_)_2_), 1.06 (d, 3H, *J* = 6.5, CH_3_), 1.02 (d, 3H, *J* = 7.0, CH_3_); ^13^C-NMR: 166.43, 138.29, 135.58, 133.21, 132.24, 131.01, 130.06, 129.87, 126.98, 126.64, 126.41, 55.95, 38.18, 30.84, 19.73, 17.55; HRMS electrospray ionization (ESI) *m*/*z* calcd. for [M+H]^+^ C_18_H_19_NCl^+^ = 284.12005, found 284.11966 (mass error ∆m = −1.39 ppm).

1-benzyl-3-isopropyl-3,4-dihydroisoquinoline (**5d**), yellow oil, 72% yield, ^1^H-NMR: 7.28–7.33 (m, 3H), 7.18–7.23 (m, 2H), 7.09–7.11 (m, 2H), 7.02–7.03 (m, 2H), 4.05–4.10 (m, 1H, CH), 3.47 (q, 2H, *J* = 16.1, CH_2_), 2.76 (dd, 1H, *J* = 14.2, 5.9, CH_2_), 2.51 (dd, 1H, *J* = 14.2, 8.3, CH_2_), 1.65–1.72 (m, CH(CH_3_)_2_, 1H), 0.89 (d, 3H, CH(CH_3_)_2_, *J* = 6.4), 0.79 (d, 3H, CH(CH_3_)_2_, *J* = 6.8); ^13^C-NMR: 170.35, 138.20, 134.95, 129.47, 129.14, 128.35, 127.31, 126.28, 55.05, 43.99, 37.81, 30.77, 19.43, 17.57; HRMS electrospray ionization (ESI) *m*/*z* calcd. for [M+H]^+^ C_19_H_22_N^+^ = 264.17468, found 264.17433 (mass error ∆m = −1.31 ppm).

### 2.2. In Silico Pharmacokinetic Profiling and Toxicity Analysis

#### 2.2.1. Theoretical Prediction of Pharmacokinetic Parameters—Absorption, Distribution, Metabolism, and Excretion (ADME) Properties

Physicochemical and pharmacokinetic parameters, and drug-likeness of SQ were analyzed using SwissADME, a free web tool [19].

#### 2.2.2. Theoretical Prediction of Toxicity

The ProToxII web tool was used to predict acute and organ toxicity, toxicity class, and LD_50_ for the compound [20,21]. 

#### 2.2.3. PASS Online Predictions

A computer-based PASS online program was used to predict biological activities of the compound, based on its structural formula [22,23,24,25]. 

### 2.3. Smooth Muscle Activity

#### 2.3.1. Ex Vivo Experiments on Gastric Smooth Muscle Preparations (SMPs) from Wistar Rats

Wistar rats (270 ± 15 g body weight, male, 11 weeks of age) were purchased from the Medical University Plovdiv, Animal Laboratory, Bulgaria. The experimental procedures were conducted with the highest confidence, adhering to the Guidelines for the Care and Use of Laboratory Animals, Medical University of Plovdiv, Bulgaria, permit No. 229/09 April 2019. The procedures were approved by the current European regulations (86/609/EEC), complied with EU Directive 2010/63/EU, and performed in strict accordance with the current guidelines of Institutional Animal Care Bulgaria. 

Four circular pieces (9–11 mm in length, 1.0–1.2 mm in width) of muscle tissue were taken from one rat stomach, with the mucosa remaining intact. As indicated by n, the samples were acquired under the following conditions: 4 °C temperature and continuous irrigation of tissues with pre-aerated (95% O_2_ and 5% CO_2_) preparation solution containing NaCl/KCl/CaCl_2_ in a ratio of 27.2/1.1/1 [25]. The samples were equilibrated in a typical physiological salt Krebs solution containing 140.0 mM NaCl, 5.0 mM KCl, 1.2 mM MgCl_2_, 1.8 mM CaCl_2_, 23.8 mM NaHCO_3_, and 11.1 mM glucose at a resting tension of 10 mN (1 g). Before each experiment, the pH of the solution was determined using an HI5521 pH meter (Hanna Instruments, Smithfield, RI, USA). The bath solution was saturated at 37 °C, pH 7.4, with a mixture of 95% O_2_ and 5% CO_2_. 

#### 2.3.2. Method for Studying the Mechanical Activity of Isolated SMPs

Experiments were performed to investigate the cumulative dose-dependent relaxation effects of compounds **5a–d** on gastric SMPs, pre-contracted with CCh (1 × 10^−^^7^ mol/L) using the previously described method [25].

### 2.4. Ethics Statement

The experiments were approved by the Ethical Committee of the Bulgarian Food Agency with permit No. 229/09.04.2019 and were carried out following the guidelines of European Directive 2010/63/EU. The animals were provided by the Medical University-Plovdiv (Animal House, Plovdiv, Bulgaria).

### 2.5. DPPH Free Radical Scavenging Assay

The DPPH free radical scavenging activity was evaluated by applying Docheva‘s method [26].

### 2.6. Statistical Analysis

For the analysis of variance, the InStat v. 3.10 (GraphPad Software, Inc., San Diego, CA, USA) computer program was utilized. Each group’s mean and standard error of the mean were determined. To compare various groups with their corresponding controls for repeated measurements, a two-way ANOVA was employed. A significant difference was deemed to be represented by a *p*-value of less than 0.05. The statistical software IBM SPSS Statistics v. 26 was utilized for the analyses.

## 3. Results and Discussion

**Figure 1:**
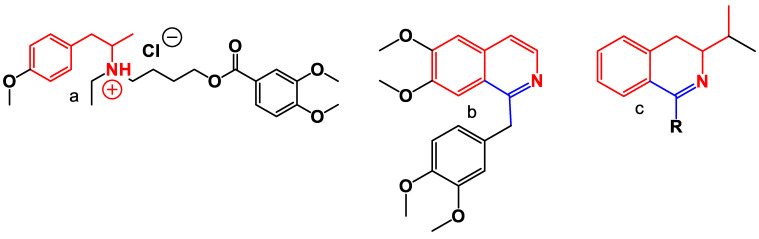
Structures of mebeverine (**a**), papaverine (**b**), and target 3-isopropyl-3,4-dihydroisoquinolines (**c**).

Abdominal pain and difficulty voiding are two of the complex symptoms of IBS. For many years, the anticholinergic spasmolytic mebeverine has been used to treat IBS. In addition, it functions as a musculotropic drug, calcium channel blocker, and an antispasmodic, and it has regulatory effects on bowel function [27]. Mebeverine, as a second-generation papaverine analog (Figure 1), reduces the excessive contractility of smooth muscle (SM) cells, giving it direct myolytic activity. 

Finding novel therapeutic strategies to influence established pharmacological targets or validate novel compounds with an alternate mode of action for IBS remains a top objective. Recently, we described the synthesis of 1-(3,4-dimethoxyphenyl)propan-2-amides and 3-methyl-1-phenylbutan-2-amides, their SM relaxant, and bioelectrical activities, and their influence on cognitive functions [17,28]. In the current work, we considered the synthesis of 1,3-disubstituted 3,4-dihydroisoquinolines due to their structural similarity to mebeverine and papaverine and their pharmacological properties as antispasmodics. 

### 3.1. Synthetic Method

Our strategy is based on the synthesis of 1-(3,4-dimethoxyphenyl)propan-2-amine and 3-methyl-1-phenylbutan-2-amine [17,28] from a starting ketone and its acylation with acid halides to corresponding amides. 

**Scheme 1:**
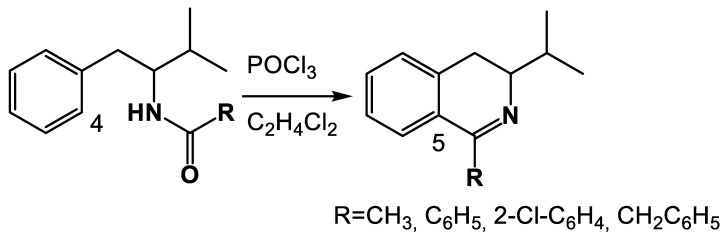
Cyclization of amides **4** to 1,3-disubstituted-3,4-dihydroisoquinolines **5**.

Next, we turned our attention toward the incorporation of acyl residue into N-heterocycles using the Bischler–Napieralski reaction to generate the isoquinoline core (Scheme 1). We found that the application of amide **4** in the Bischler–Napieralski reaction with phosphorus(V)oxychloride in 1,2-dichloroethane and a reflux for 1–3 h yields the corresponding 1-substituted 3-isopropyl products **5a–d** in moderate yields. The NMR data confirmed the structure of the obtained compounds (Supplementary Materials Figures S1–S8). 

### 3.2. In Silico Predictions

Using in silico methods, the drug-likeness of the compounds and their ADMET properties have also been predicted theoretically. Increased lipophilicity, membrane permeability, and pharmacological activity are the outcomes of the conformational restriction of small drug molecules brought about by the incorporation of this noncovalent interaction into drug design [29]. 

According to Lipinski’s rule of five [30], the ADME investigation demonstrates that the compounds have good gastrointestinal absorption and can pass across the blood–brain barrier. We found that **5a–d** met the standard values (Table 1) of molecular weight (MW ≤ 500), XLOGP3 ≤ 5, ESOL or estimated solubility (logS: not more than six), saturation (Fraction Csp^3^ or fraction of carbons in the sp^3^ hybridization: not less than 0.25), and flexibility (RB: no more than nine). 

A Csp^3^ of 0.28–0.46 and 1–3 rotatable bonds satisfied the standard value of the drug-likeness concept. The compounds scored a bioavailability 0.55, which relates to good oral absorption [31]. They also had a good synthetic accessibility score (3.12–3.63), which is a very important parameter in the drug discovery process. Pharmacological toxicity is a consequence of cytochrome P450 (CYP) inhibition [32,33]. The studied compounds are not predicted as P-glycoprotein substrates. The calculations showed that **5b** and **5d** are expected to inhibit the CYP1A2, CYP2D6, and CYP3A4 isoforms, while 5a and 5c are expected to inhibit the CYP2D6 and CYP3A4 isoforms. The compounds have a log Kp between −4.28 and −5.31, which indicate good skin permeability. 

Acute toxicity data (Pro Tox-II) indicated that the toxicity class of all the compounds is class 3. The LD_50_ predicted for all the compounds is 240 mg/kg. **5b** and **5d** were predicted to have mutagenicity (0.55% probability), whereas other organ toxicities were absent.

As a result, all the synthesized compounds can be evaluated as potential therapeutic candidates. The PASS Online Program predicted the muscle relaxant and antioxidant activities of the synthesized compounds. Once synthesized, we turned our attention to investigating the biological activities of the synthesized 3,4-dihydroisoquinolines. 

### 3.3. Spasmolytic Activity

Traditionally, smooth, skeletal, and cardiac muscles have been categorized as excitable tissues with phenotypic variety, capable of contracting or relaxing in response to endogenous or external pharmacological agents. Since smooth muscle contractile failure is a characteristic feature of many disorders, in the current paper, we investigated the parameters of the spontaneous contractile responses of smooth muscle cells.

There are two types of smooth muscle: tonic and phasic. Vascular dilatation occurs in tonic smooth muscles. The gastrointestinal and urogenital systems are home to phasic smooth muscles, which, as their name implies, contract rhythmically. The frequency, amplitude, and strength of the tonic response define the phasic contraction of phasic smooth muscles, which operate as if they are solitary muscles. Interstitial cells of Cajal, which are pacemaker cells, can start phasic contractions, and they can be sped up or slowed down by turning on an electromechanical or pharmacomechanical coupling. These processes can be modulated by muscle-specific signals depending on the type of muscle, its function, and the amount of force required. The force in pharmacomechanical coupling is triggered by receptor signaling, which may or may not entail a change in the membrane potential. In electromechanical coupling, the force is triggered by a change in the membrane potential [34].

This distinction is important when examining the effects of freshly produced pharmacological drugs or their precursors and analogs on smooth muscle function ex vivo, as all smooth muscles can be stimulated by both methods. Early research on smooth muscle in animals (dogs and rabbits) led to the distinction between pharmacomechanical coupling, which produces action potentials and phasic contractile activity, and electromechanical coupling, which results in graded depolarizations and contractions [35].

It is currently understood that vasoconstrictor signals cause the smooth muscle contraction through two mechanisms: (1) calcium influx, which activates myosin light chain kinase (MLCK); and (2) second messengers, which inhibit the myenteric plexus (MP) and make myofilaments more sensitive to calcium activation [36]. These mechanisms are active at cytosolic Ca^2+^ levels of the order of 10^−5^ M, according to Droogmans and Casteels [37]. Vasodilators, on the other hand, relax smooth muscle by desensitizing myofilaments to calcium by blocking calcium influx and activating the MP. Mebeverine is a well-known medication with direct musculotropic activity that involves Ca^2+^ ion exchange and excitable membrane stability [38]. It is a well-known drug that has been shown to help with irritable bowel syndrome, but it also has a lot of unfavorable side effects [39].

Thus, it makes sense and supports our scientific purpose to compare the myotropic effect of mebeverine with recently reported compounds that have a comparable structure through a functional analysis of their activating and inhibiting abilities. In a prior investigation, we used the single sucrose gap approach to determine these bioelectrical effects for the isoquinoline precursors [17]. In the present work, we compared the spasmolytic activity of 3,4-dihydroisoquinolines **5a–d** to their previously described precursors **4a–d**.

Antispasmodics can be categorized into two groups based on their mechanism of action: neurotropic drugs, which block nerve impulses from reaching smooth muscle cells and alter muscle bioelectric activity; and myotropic drugs, which have an impact on the biochemical processes that control smooth muscle contractions. 

The myotropic action in all muscle cells is expressed as contraction or relaxation and is dependent on changes in the concentration of cytosolic calcium [40].

**Figure 2:**
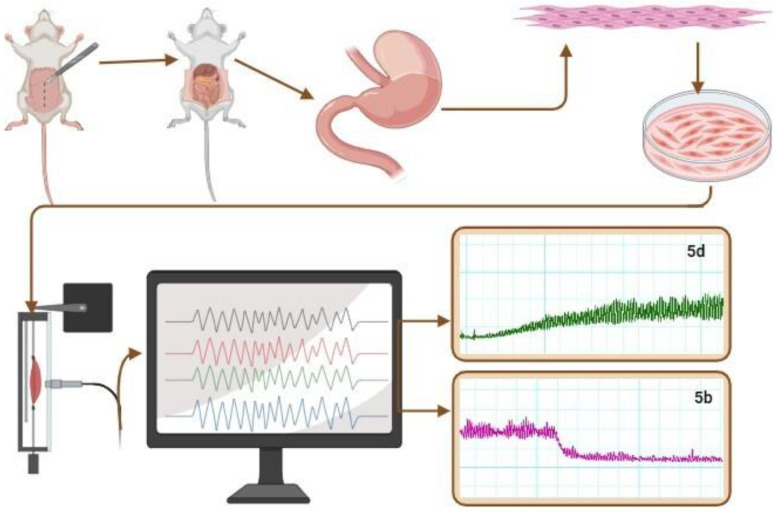
A graphical depiction outlining the procedure for examining the mechanical functions of isolated smooth muscle preparations. This includes illustrative traces for compound **5d** compared to **5b** and was generated using BioRender.com (accessed on 29 January 2024).

SMPs from the central region of the rat stomach were studied in order to ascertain the biological effects of compounds **4a–d** and **5a–d** on the mechanical contractile activity of smooth muscle and to assess the effects of induced relaxation or contraction (Figure 2, Table 2). As a control, the response of SMPs to mebeverine was observed. This decision was influenced by the fact that the characteristics of the spontaneous contractile activity (SCA) of smooth muscle strips were unaffected by mebeverine for gastrointestinal disorders when it was supplied in the tissue bath at a concentration of 5 × 10^–5^ M.

**Figure 3:**
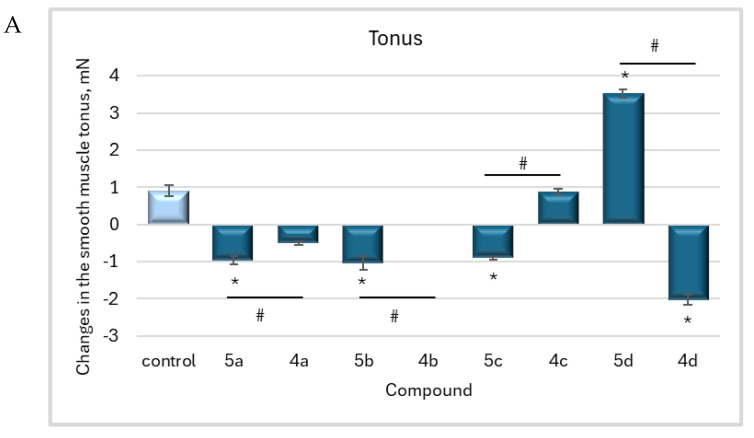

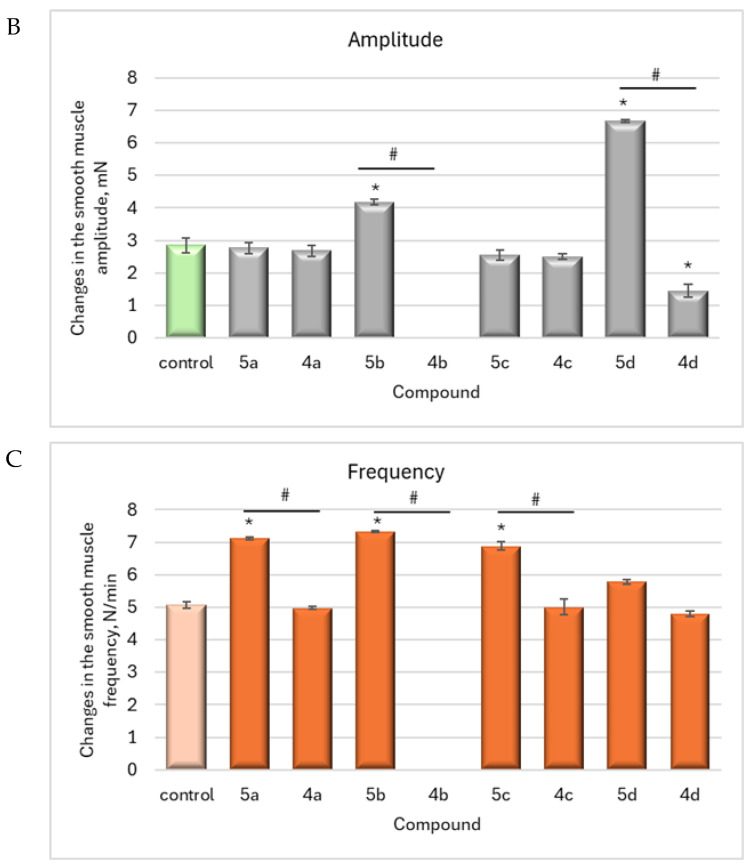
Changes in the smooth muscle tissue’s (**A**) tonus, (**B**) amplitude, and (**C**) frequency induced by compounds **4a–d** compared to those of **5a–d** (5 × 10^−5^ mol/L) (*n* = 12). Statistically significant differences: * *p* < 0.05, comparison is between the measured value and mebeverine as a control; ^#^ *p* < 0.05, comparison is between the measured values of precursors to isoquinolines, **4a** vs. **5a**, **4b** vs. **5b**, **4c** vs. **5c**, and **4d** vs. **5d**.

Time efficiency was measured by observing all mechanical changes for 60 min under identical experimental conditions, with the investigated chemicals acting at a concentration of 5 × 10^−5^ M. We can classify substances **4a–d**, **5a**, and **5c** as fast-acting as they reached their maximal biological potential within 4 min. Since **5b** and **5d** only became fully effective after 10 min, they can be classified as having a longer delayed action. The tonus component, frequency, and amplitude of contraction for **5b** (Figure 3), as well as the tone and frequency of contraction, were reliably reduced in the isometric registration of the evoked smooth muscle responses [40]. However, the amplitude of the single contractions under impact with **5a** and **5c** remained unaffected. A relaxation effect was found for **4d**; however, its tone and amplitude statistically changed (Table 2).

The SMPs completely relaxed and the contractions stopped in the background of compound **4c**, designating it as a prototype SM relaxant. The induced relaxations can be attributed to the stimulation of calcium uptake, the inhibition of calcium influx, a direct decrease in intracellular Ca^2+^ concentration, or the inhibition of calcium release from intracellular storage sites [41]. The current paper aimed to determine the mechanism of these effects in the isolated intestinal SMPs.

Contrary to the mechanisms of the relaxation with substances **4b**, **4d**, **5b**, **5a**, and **5c**, substances **4a** and **4c** did not exhibit any visible effect on the mechanical properties and contraction force of the SMPs, even at higher concentrations (up to 10^−4^ M), as determined by the limitations of the applied SM model in the ex vivo study.

The reported stimulating impact of compound **4d** was unexpected given the software estimates and relaxation effects we confirmed for some of the tested compounds. Sharp, strong, and consistent in both amplitude and tone, the contraction likely impacts the activation of calcium-delivering contractile proteins or increases the flow of Ca^2+^ into the intracellular space (Table 2 and Figure 3).

Finally, signal transduction is essential for contractile cell function. We found that compound **5b** demonstrates considerable relaxation by altering the primary parameters of tone and frequency of the SCA of gastric SMs. Substance **5d**, in its capacity as a stimulating signal, likely influences calcium translocation, which promotes tonic and rhythmic muscular activity. These data give us a reason to identify the newly synthesized substances, especially **5b** and **5d**, as essential modulating regulators and potentially use them as therapeutic molecules.

### 3.4. DPPH Radical Scavenging Activity

Finally, the in silico methods predicted antioxidant activity for the compounds, and we evaluated the antioxidant potential in vitro using a DPPH radical scavenging assay [26] using quercetin and rutin as standards. We found that only compound **5d** showed significant antioxidant potential compared to the standards. 

**Figure 4:**
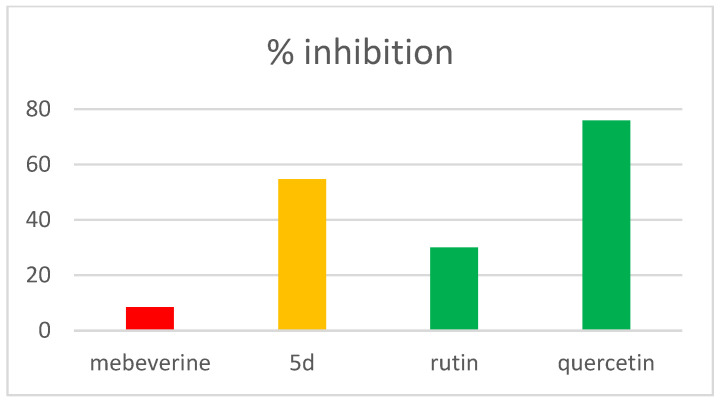
Antioxidant activity of **5d** compared to those of mebeverine, rutin, and quercetin as positive controls.

The obtained data showed that **5d** at a concentration of 31 ± 0.25 µM exhibited a percentage inhibition of 55%, which was compared to the antioxidant activities of quercetin (31 µM with 75.9% inhibition), rutin (31 µM with 30% inhibition), and mebeverine (31 µM with 8% inhibition) (Figure 4). The results showed that **5d** has better antioxidant activity than rutin and mebeverine, but lower than that of quercetin. 

## 4. Conclusions

A number of novel 3-isopropyl 3,4-dihydroisoquinolines were synthesized as potential antispasmodics. All the synthetic compounds, in general, displayed favorable expected in silico profiles and were identified as potentially useful oral medications with low toxicity. Isolated tissues were used to demonstrate their ex vivo spasmolytic activity compared to those of previously described precursors. The results demonstrate that compound **5d** can act as a contractile agent in its capacity as a stimulating signal, probably influencing calcium translocation, which promotes tonic and rhythmic muscular activity, and compound **5b** exhibits significant relaxation by changing the primary parameters of tone and frequency of the SCA of gastric SMs. Compound **5d** also shows very good antioxidant activity, better than mebeverine and rutin but lower than that of quercetin. 

Thus, the newly synthesized compounds **5b** and **5d** were found to have favorable spasmolytic activity and could be fair and successful options for the long-term oral therapy of chronic IBS, according to the summarized results of the conducted trials. Therefore, it is necessary to conduct additional in vivo research in the future to explore the potential therapeutic uses of these 3,4-dihydroisoquinolines.

## Figures and Tables

**Table 1:** ADME parameters of compounds **5a–d** representing drug-likeness.

Compound	MW, g/mol	XLOGP3	ESOL logS	Fraction Csp^3^	RB	BA Score	SA Score	LD_50_, mg/kg
**5a**	187.28	3.01	−3.15	0.46	1	0.55	3.12	240
**5b**	249.35	4.66	−4.66	0.28	2	0.55	3.49	240
**5c**	283.8	5.29	−5.24	0.28	2	0.55	3.53	240
**5d**	263.38	4.60	−4.62	0.32	3	0.55	3.63	240

MW: molecular weight, XLOGP3: octanol/water partition coefficient, ESOL logS: estimated aqueous solubility, fraction Csp^3^: ratio of sp^3^-hybridized carbons over the total carbon count, RB: rotatable bonds, BA: bioavailability, SA: synthetic accessibility, predicted LD_50_: lethal dose to 50% of animals.

**Table 2:** Changes in the parameters of SCA of gastric SMPs under the influence of **4a**–**d** compared to **5a**–**d**. All data are expressed as mean values ± standard error of the mean.

SCA Parameters	Control	5a	4a	5b	4b	5c	4c	5d	4d
tonus, mN	0.91 ± 0.16	−0.96 ± 0.11	−0.50 ± 0.06	−1.04 ± 0.19	0	−0.91 ± 0.04	0.88 ± 0.08	3.53 ± 0.11	−2.03 ± 0.13
frequency, min^−1^	5.07 ± 0.09	7.12 ± 0.05	4.98 ± 0.05	7.33 ± 0.03	0	6.88 ± 0.13	5.01 ± 0.24	5.78 ± 0.07	4.79 ± 0.08
amplitude, mN	2.85 ± 0.22	2.77 ± 0.17	2.68 ± 0.18	4.18 ± 0.08	0	2.55 ± 0.16	2.50 ± 0.09	6.66 ± 0.04	1.45 ± 0.20

## Data Availability

The original contributions presented in the study are included in the article/supplementary material, further inquiries can be directed to the corresponding author.

## Supplementary Materials

The following supporting information can be downloaded at https://www.mdpi.com/article/10.3390/biomedicines12071556/s1, Figure S1: ^1^H-NMR spectrum of compound **5a**, Figure S2: ^13^C-NMR spectrum of compound **5a**, Figure S3: ^1^H-NMR spectrum of compound **5b**, Figure S4: ^13^C-NMR spectrum of compound **5b**, Figure S5: ^1^H-NMR spectrum of compound **5c**, Figure S6: ^13^C-NMR spectrum of compound **5c**, Figure S7: ^1^H-NMR spectrum of compound **5d**, Figure S8: ^13^C-NMR spectrum of compound **5d**.
