# Supplementary material for: Spasmolytic Activity of 1,3-Disubstituted 3,4-Dihydroisoquinolines
Source: Biomedicines. 2024 Jul 13;12(7):1556. doi: 10.3390/biomedicines12071556 (PMC11275145; doi:10.3390/biomedicines12071556)

# Spasmolytic activity of 1,3-disubstituted 3,4-dihydroisoquinolines

**Miglena Milusheva, Mihaela Stoyanova, Vera Gledacheva, Iliyana Stefanova, Mina Todorova, and Stoyanka Nikolova**

**Table of Contents:**

Figure S1: $^{1}$H-NMR spectrum of compound **5a**, page 2, 3

Figure S2: $^{13}$C-NMR spectrum of compound **5a**, page 4

Figure S3: $^{1}$H-NMR spectrum of compound **5b**, page 5

Figure S4: $^{13}$C-NMR spectrum of compound **5b**, page 6

Figure S5: $^{1}$H-NMR spectrum of compound **5c**, page 7

Figure S6: $^{13}$C-NMR spectrum of compound **5c**, page 8

Figure S7: $^{1}$H-NMR spectrum of compound **5d**, page 9

Figure S8: $^{13}$C-NMR spectrum of compound **5d**, page 10

Figure S1: $^{1}$H-NMR spectrum of compound **5a**

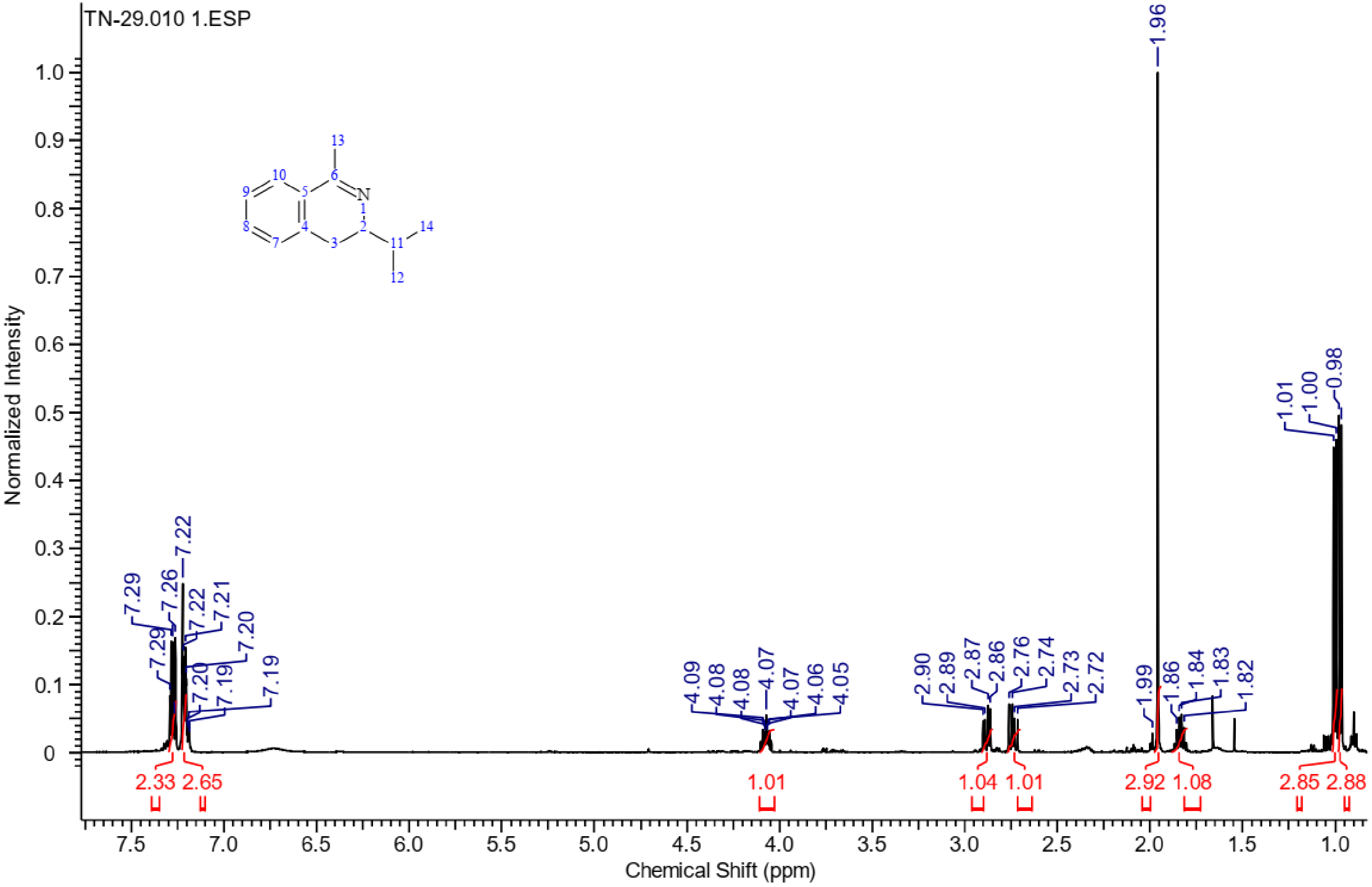

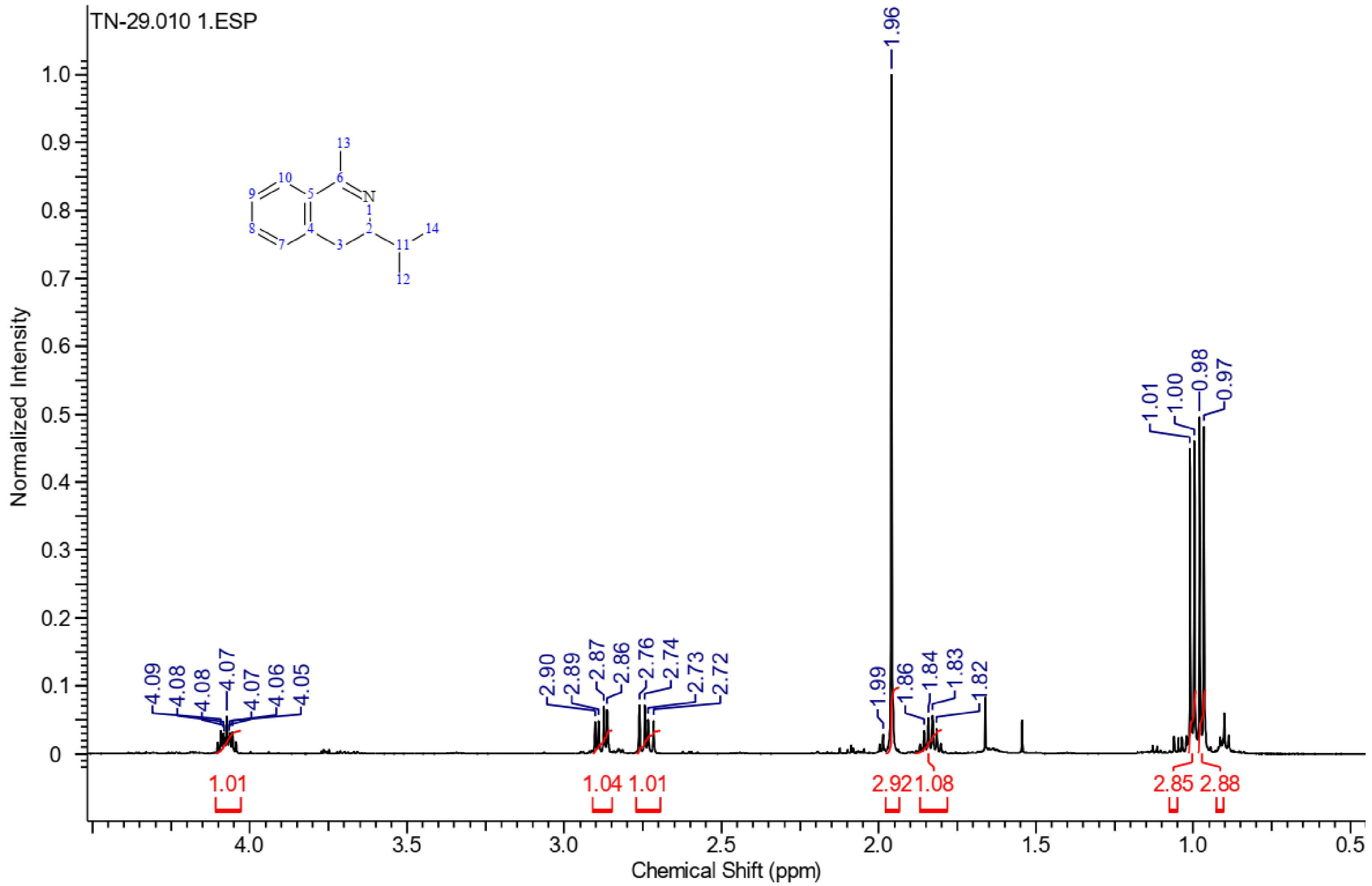
TN-29.010 1.ESP
Normalized Intensity
Chemical Shift (ppm)
4.09 4.08 4.08 4.07 4.07 4.06 4.05
2.90 2.89 2.87 2.86 2.76 2.74 2.73 2.72
1.99 1.96 1.86 1.84 1.83 1.82
1.01 1.00 0.98 0.97
1.01 1.04 1.01 2.92 1.08 2.85 2.88
N

Figure S2: $^{13}$C-NMR spectrum of compound **5a**

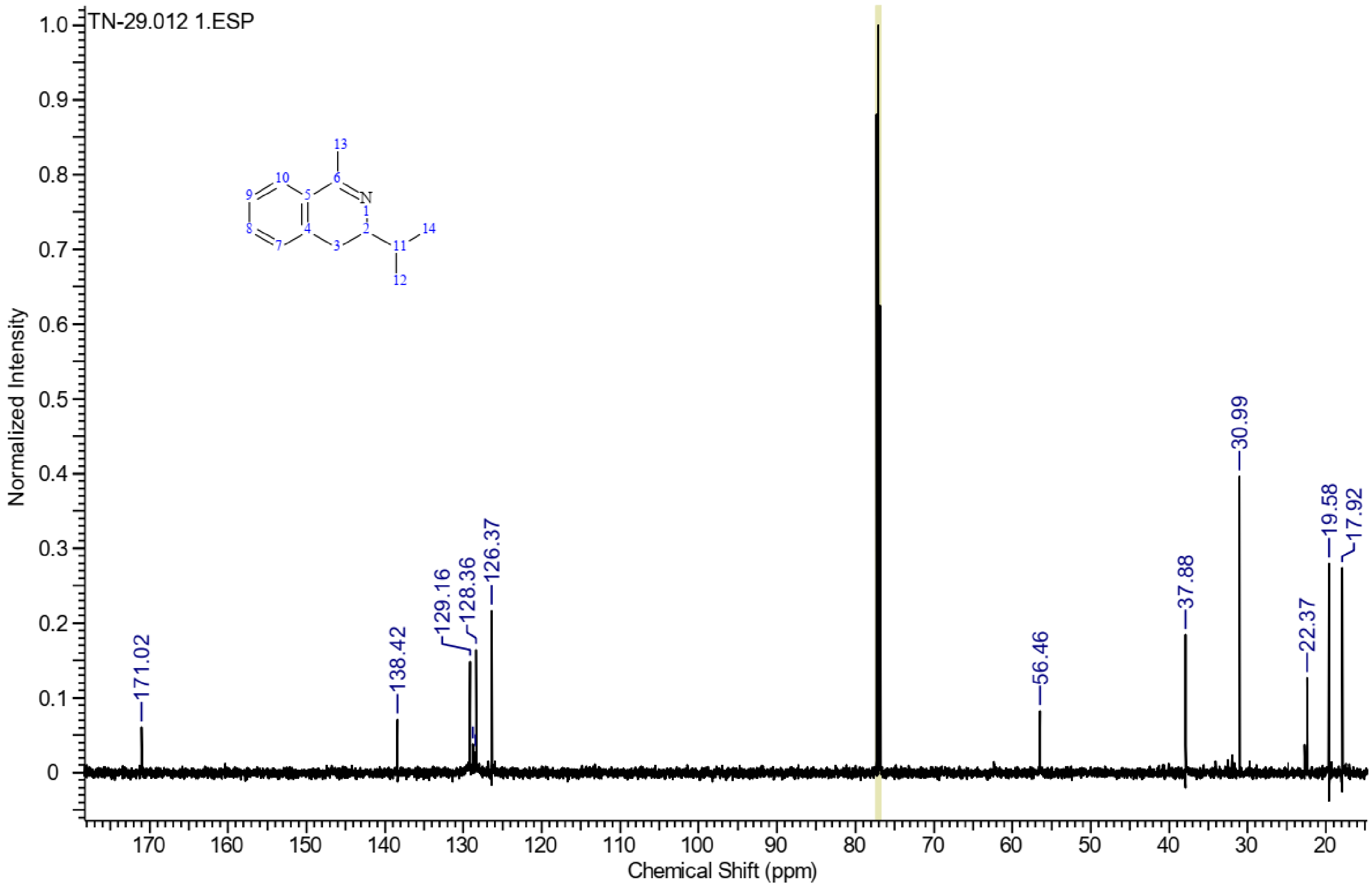

Figure S3: $^{1}$H-NMR spectrum of compound **5b**

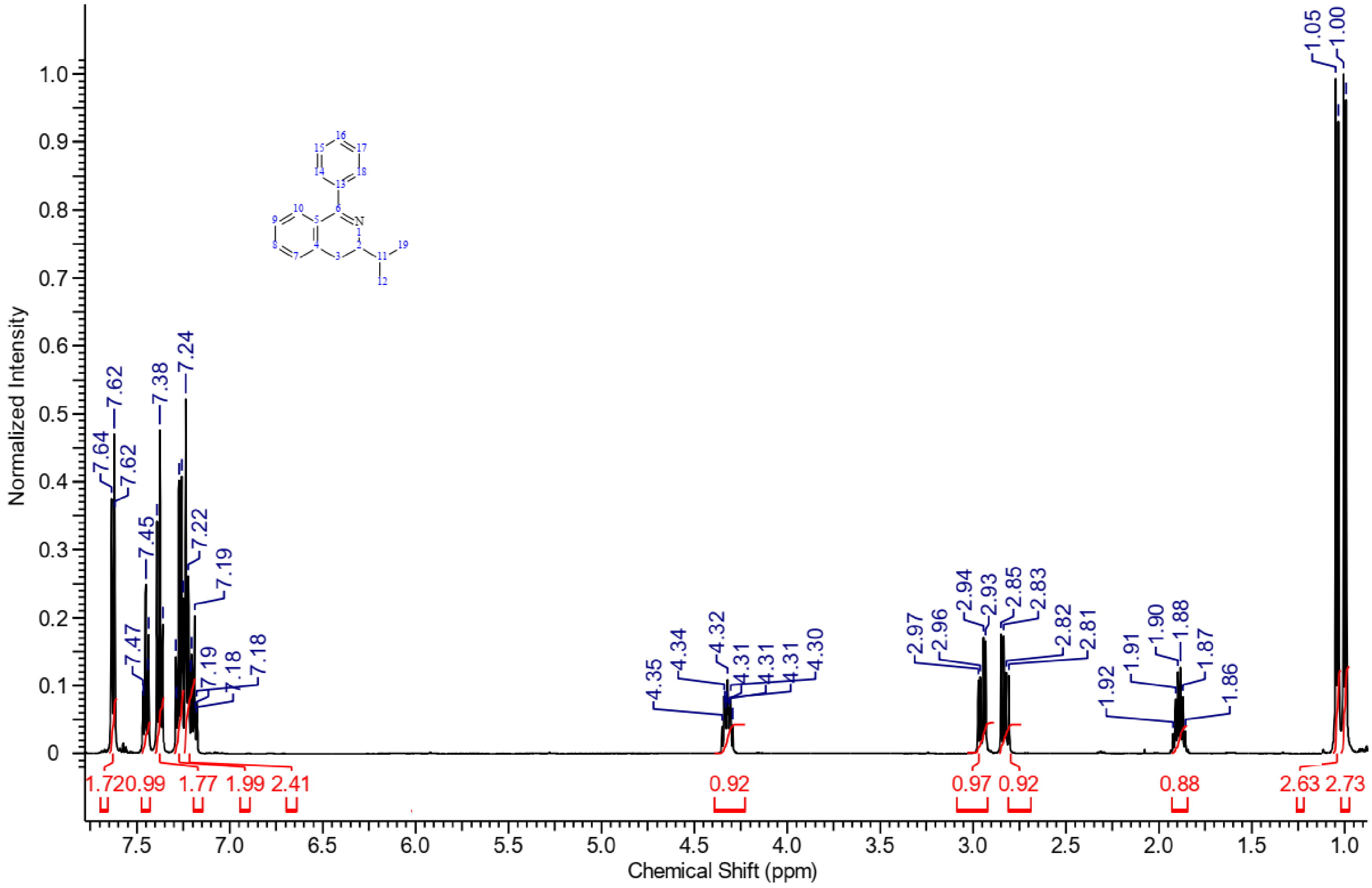

Figure S4: $^{13}$C-NMR spectrum of compound **5b**

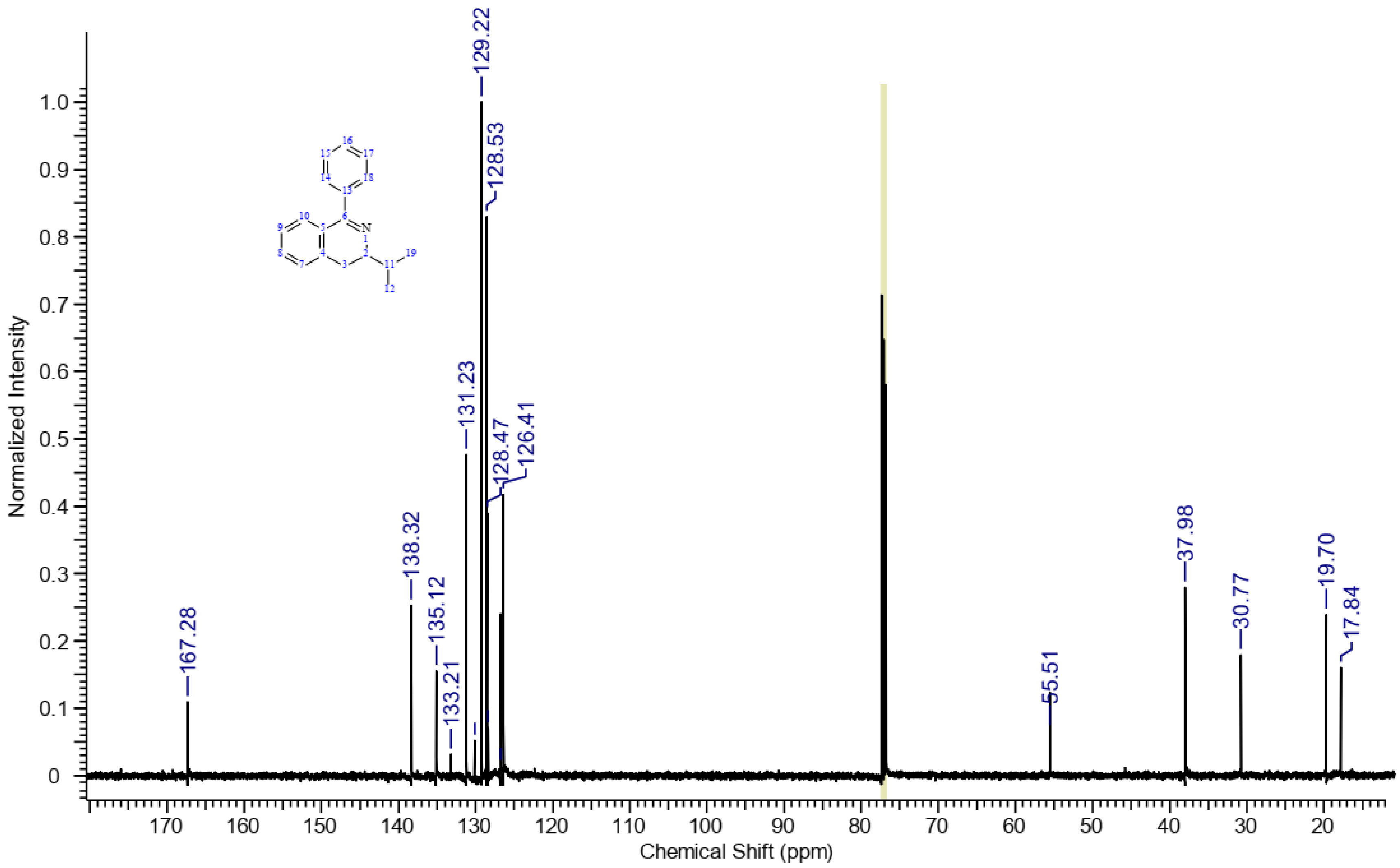

Figure S5: $^{1}$H-NMR spectrum of compound **5c**

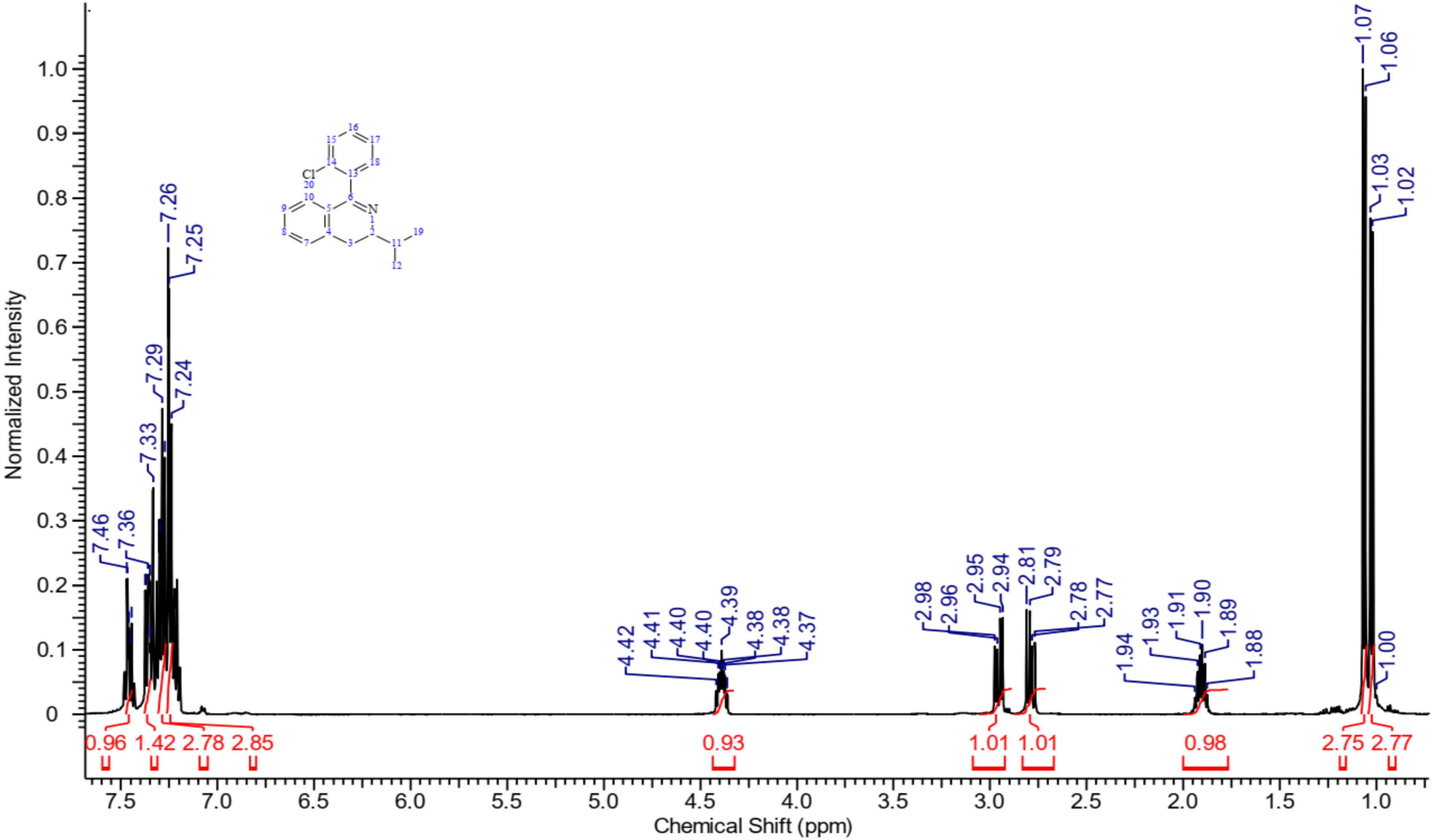

Figure S6: $^{13}$C-NMR spectrum of compound **5c**

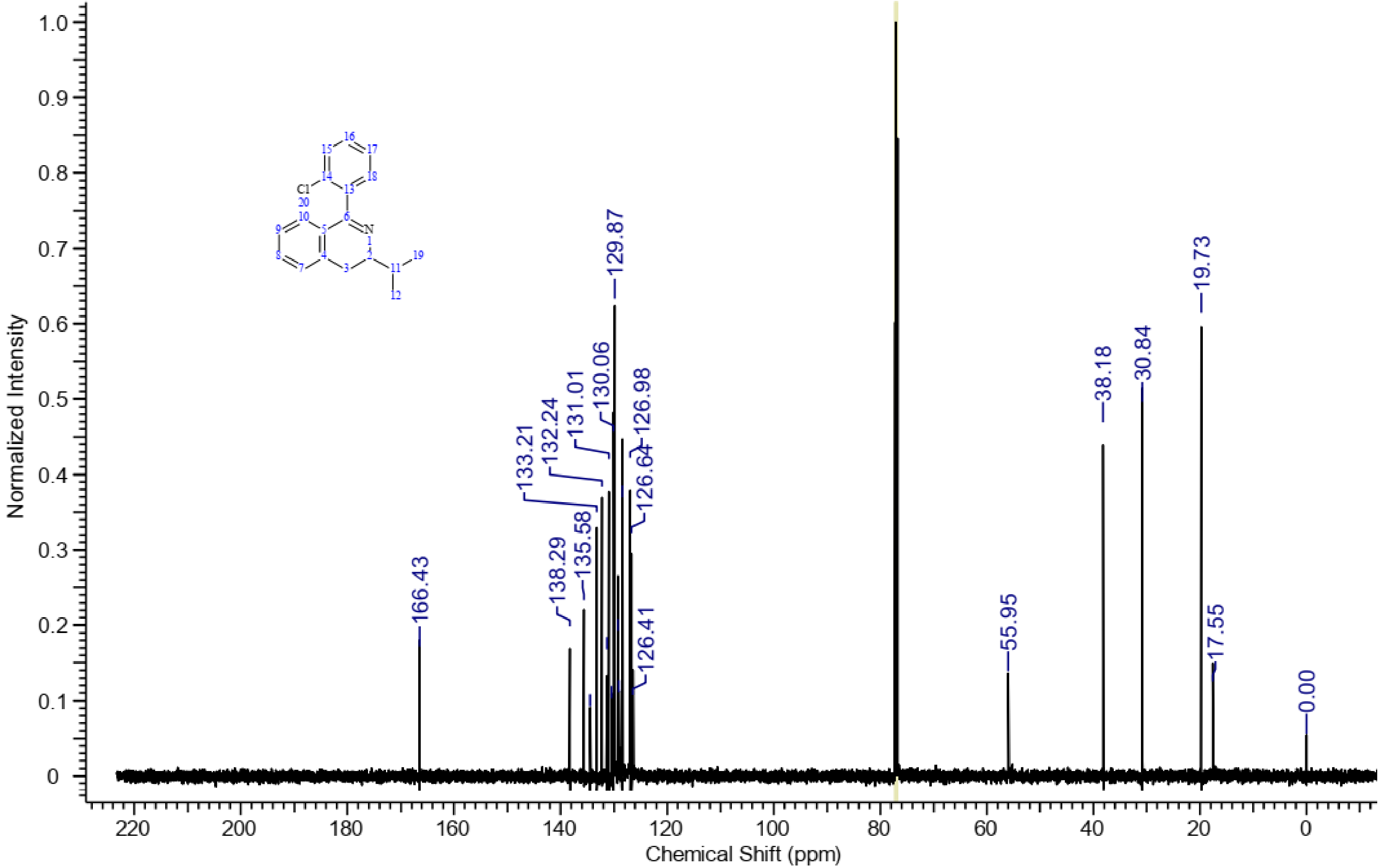

Figure S7: $^{1}$H-NMR spectrum of compound **5d**

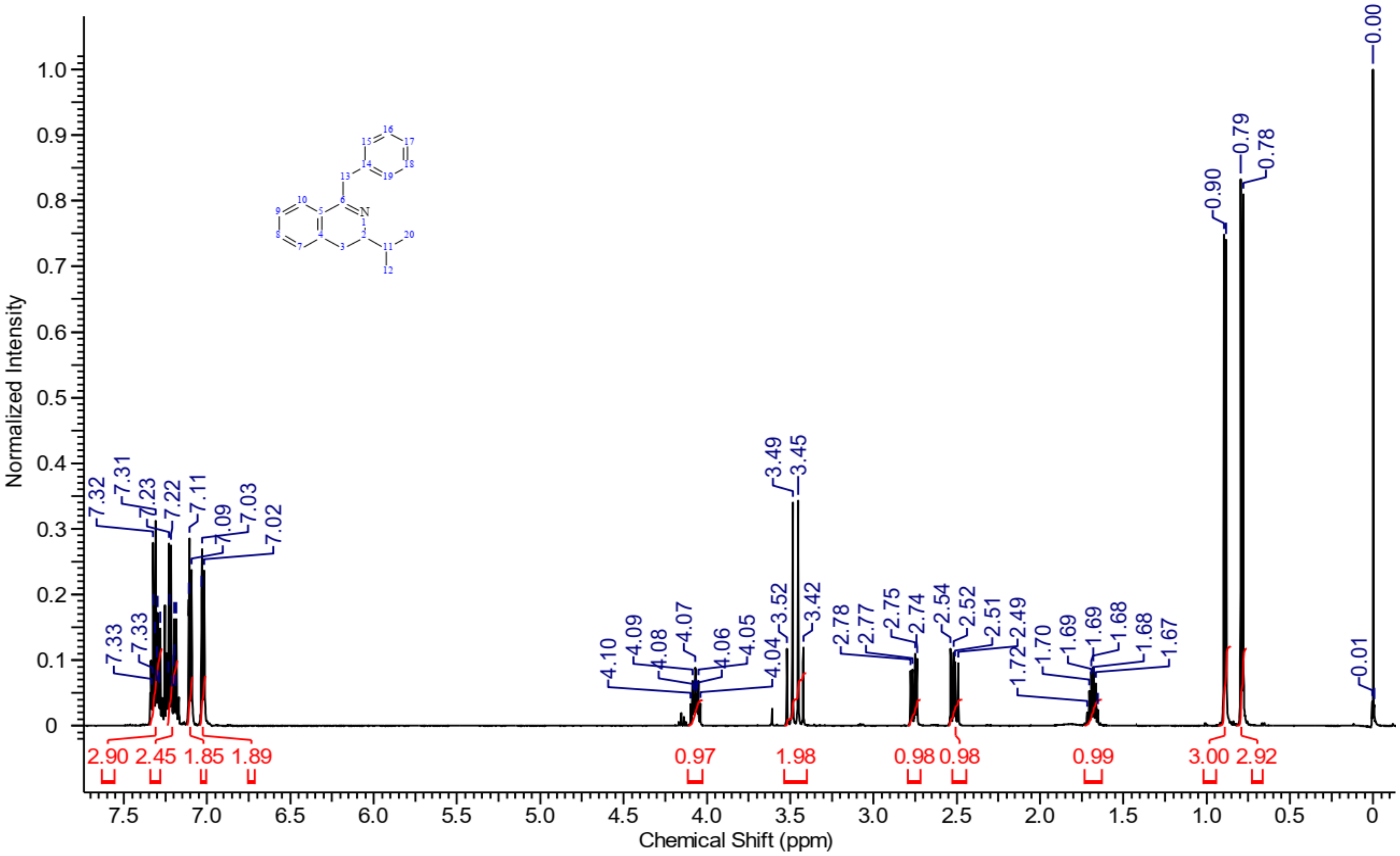

Figure S8: $^{13}$C-NMR spectrum of compound **5d**

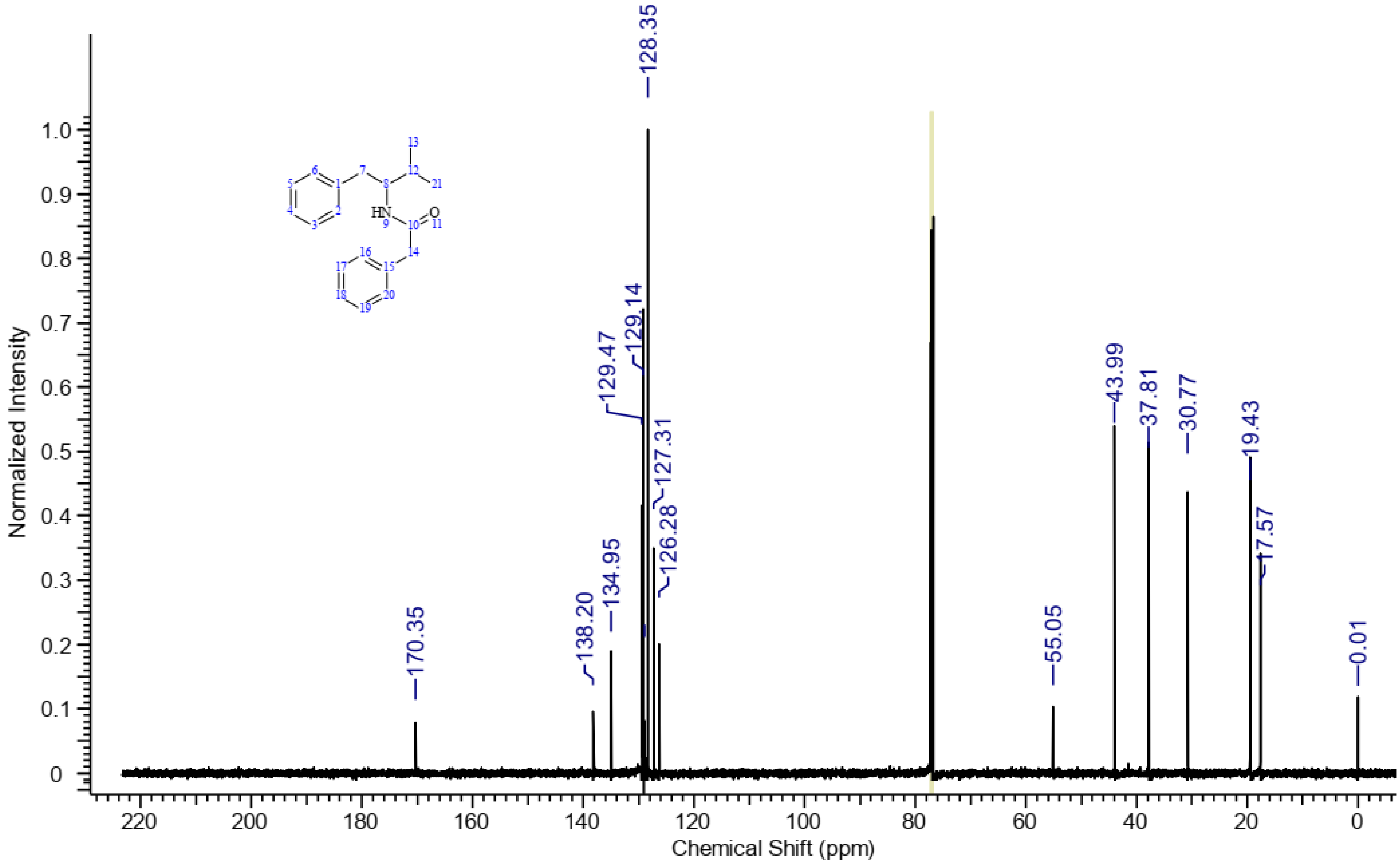

Supplement: Supplementary file 1 [file biomedicines-12-01556-s001.zip › biomedicines-3074789-supplementary.pdf]
